# The Influence of Various Morphologic and Hemodynamic Carotid Plaque Characteristics on Neurological Events Onset and Deaths

**DOI:** 10.1100/tsw.2009.74

**Published:** 2009-06-30

**Authors:** Milan D. Brajović, Nataša Marković, Goran Lončar, Nikola Šekularac, Dejan Kordić, Nebojša Despotović, Predrag Erceg, Branislav Donfrid, Zvezdan Stefanović, Milica Bajčetić, Ljiljana Brajović, Živorad Savić

**Affiliations:** ^1^ Cardiology Department, Zvezdara University Hospital, Belgrade, Serbia; ^2^ Clinical Department for Geriatrics, Zvezdara University Hospital, Belgrade, Serbia; ^3^ Vascular Surgery Department, Zvezdara University Hospital, Belgrade, Serbia; ^4^ Department of Pharmacology, School of Medicine, University of Belgrade, Serbia; ^5^ Civil Engineering Faculty, Department of Technical Physics, Belgrade, Serbia; ^6^ Institute of Radiology, Clinical Centre of Serbia, Belgrade, Serbia

**Keywords:** carotid stenosis, carotid plaque, cerebrovascular disorders, neurological symptoms, stroke, ultrasonography, angiography, X-ray computed tomography, color duplex scanning, peak flow velocities

## Abstract

A group of 72 patients with 111 asymptomatic carotid stenoses (ACS), mean age 65.42 ± 9.21, and a group of 36 patients with 58 symptomatic carotid stenoses (SCS), mean age 67.63 ± 8.79, were analyzed prospectively during a 3-year follow-up period. All patients underwent color duplex scan sonography (CDS), carotid arteriography, computed tomography (CT) scan, and neurological examination. The aim of the study was to analyze the correlation between echo plaque morphology (degree and plaque quality), local hemodynamic plaque characteristics, ischemic CT findings, and onset of new neurological events and deaths. The results analysis showed significantly more ACS in the group of 30–49% stenosis (*p* < 0.001), but significantly more SCS in the group of 70–89% (*p* < 0.0001) and ≥90% stenosis (*p* < 0.05). Fibrous plaque was more frequent in the ACS group (*p* < 0.001), while ulcerated and mixed plaques were more frequent in the SCS group (both *p* < 0.0001). In the SCS group, a significantly higher frequency of increased peak systolic and end diastolic velocities was noted at the beginning and end of the study (both *p* < 0.01), as well as for contralateral common (CCA) or internal carotid artery (ICA) occlusion (*p* < 0.05 and *p* < 0.01, respectively), but reduced carotid blood flow volume (*p* < 0.05) only at the end of the study. In the ACS group, the best correlation with new neurological events and deaths was shown with positive CT findings, peak systolic flow velocity over 210 cm/sec, end diastolic flow velocity over 110 cm/sec, plaque stenosis ≥70%, plaque ulceration, mixed plaque (all *p* < 0.0001); stenosis ≥50% (*p* < 0.001); and reduced carotid blood flow volume (*p* < 0.05).

## INTRODUCTION

Nowadays, timely diagnosis and treatment of cerebrovascular ischemic disease (CVID) are among the most important medical concerns. There are several reasons for this, but the most important is the fact that stroke, mainly as a consequence of CVID, is one of the most frequent causes of death[1]. Serious disability after stroke due to irreversible damage of the brain with limited possibility for rehabilitation requires permanent care by another person. In some countries, stroke is the major health problem, with high incidence and serious social and economic consequences[2]. The unsatisfactory results of immediate and late stroke treatment require researchers to devise better neuroprotection therapies for ischemic stroke[3].

In the U.S., stroke ranks No. 3 among all causes of death, behind diseases of the heart and cancer. Among adults, ages 20 and older, the estimated prevalence of stroke in 2005 was 5,800,000, and new or recurrent stroke attacks are experienced each year by 780,000 people[4]. These data illustrate the importance of timely diagnosis and treatment in early stages of CVID.

Studies of carotid atherosclerosis[5] showed that patients with moderate or severe carotid stenosis were often without transient ischemic attacks (TIA) and were asymptomatic for a long time, but eventually experienced stroke. The Harvard Cooperative Stroke Registry presented that 74% of strokes happened without warning, and 50% of all strokes were due to carotid or vertebral artery thrombosis in patients without TIA. In 75% of ischemic strokes due to carotid atherosclerosis proved by carotid arteriography, 35% had not been preceded by TIA[6].

The fact that nearly 50% of all strokes happened due to embolus or thrombosis of extracranial segments of carotid or vertebral arteries recognizes that color duplex scanning of carotid and vertebral arteries is an appropriate and highly accurate diagnostic method[7].

The aim of this study was to analyze relationships between echo plaque morphological features (degree of the stenosis, echo plaque surface, and composition qualities), on the one hand, and local hemodynamic flow features (carotid flow reduction on the affected side, presence and influence of contralateral occlusion of internal or common carotid artery, peak systolic and end diastolic flow velocities on the stenosis site), on the other, with onset of neurological events (neurological symptoms and objective findings of neurological disorders) and deaths.

## MATERIALS AND METHODS

Seventy-two patients (pts) with 111 asymptomatic carotid stenoses (ACS) and 36 pts with 58 symptomatic carotid stenoses (SCS) were prospectively studied. Only carotid stenoses equaling 30% of the cross-sectional area or more were included in the analysis. The ACS group was composed of 49 males and 23 females, mean ages 66.7 and 62.3 years, respectively; and the SCS group included 28 males and 8 females, mean ages 68.2 and 62.4 years, respectively. In 39 pts with ACS, stenoses were present on both carotid arteries, and in 33 pts, on one carotid artery. In the SCS group, 22 and 14, respectively. ACS implied stenosis of common (CCA) or internal carotid artery (ICA) among the pts without any neurological symptoms, as well as objective neurological disorder until the moment of enrollment into the study. SCS implied patients with documented history of TIA, amaurosis fugax (AF), reversible ischemic neurological deficit (RIND), progressive (PIND) or chronic (CIND) ischemic neurological deficit attributable to carotid artery stenosis. The exclusion criteria covered pts with diagnoses of absolute arrhythmia, left ventricular (LV) or atrial thrombus, LV diastolic size exceeding 7 cm and LV ejection fraction below 30%, left atrial systolic size exceeding 5 cm, dilatative cardiomyopathy, and infective endocarditis and state after implantation of artificial heart valves.

Color duplex scan (CDS) examinations were performed in the Zvezdara University Hospital echo laboratory at the beginning of the study, after 3 and 6 months, and then in 6-month intervals to the end of the 3-year follow-up.

All scans were performed with a vascular linear 7-MHz duplex scan head, using an Acuson Aspen Advanced Ultrasound machine. All subjects were examined in the supine position with slight head extension. Degree of stenosis was calculated by the ratio of the cross-sectional area of stenosis to cross-sectional area of whole artery at the site of maximal stenosis, expressed in percents. Analysis of the plaque quality took into account the estimation of plaque surface and echo composition, using the cross-sectional and longitudinal projections obtained by the anterior, lateral, and posterolateral projections to obtain images of the plaques. Plaque quality echo features were defined as: calcified – high echoic with distal acoustic shadows; fibrous – uniformly echoic without acoustic shadows; intraplaque hemorrhage – uniformly echolucent with echoic fibrous cap on the border of the plaque; mixed (heterogeneous) – both echolucent and echoic areas in the same plaque with more than 50% echoic zone; ulceration – the discontinuation of fibrous cap presented as an interruption of its echogenity (Fig. 1).

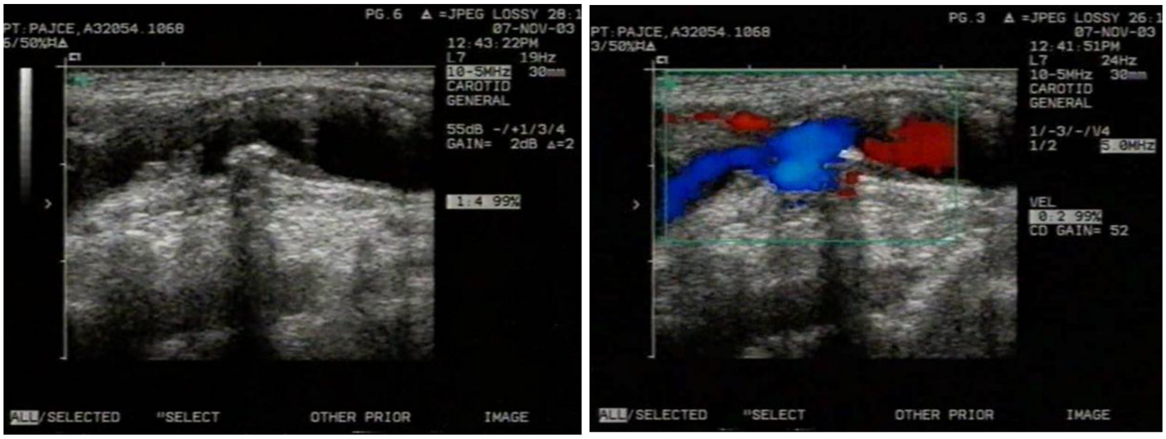

A B

**FIGURE 1**. Ulcerated plaque with big embolic potential (A). Color inflow inside of the plaque (B).

Local flow features at the site of carotid stenosis were analyzed by pulse wave color Doppler flow measurements: peak systolic velocity (PSV) and end diastolic velocity (EDV) in centimeters per second (cm/sec); blood flow volume was calculated using values of peak systolic velocity, flow velocity integral, cross-sectional area surface at the site of stenosis, and heart rate. A significant blood flow reduction was defined as the flow below 200 ml/min in the carotid communal and/or carotid internal artery. Occlusion of the contralateral CCA or ICA was defined as the absence of flow in the affected artery.

All pts underwent panarcography and selective carotidography at the beginning and end of the 3-year follow-up. A seriographic Siemens machine was used, and degree of stenosis was estimated by the Barnes method using anteroposterior, left, and right oblique projections.

All pts underwent cerebral computed tomography (CT) examination at the beginning and each year until the end of the follow-up. The examinations were performed by a CT Pace Plus General Electric machine. Ten-section planes per 10 mm from the skull baseline were analyzed – clear hypodense lesions ipsylateral with the carotid stenosis were considered as ischemic cerebral infarction (CVI).

Neurological examinations were performed by a neurologist who established types of neurological events: AF – implying transient monocular blindness; TIA – implying a brief episode of neurological disfunction, such as hemiparesis, hemiparestesia, dysarthria, dysphasia completely reversibile up to 24 h from onset; stroke – implying persistent hemiparesis or hemiplegia, with or without speech disturbances due to CVI.

To establish the presence of cardiovascular risk factors (RF), coronary artery disease (CAD), and peripheral occlusive arterial disease (POAD) on lower legs, all pts underwent the following diagnostic procedures: for CAD – selective coronarography at the beginning of the study, where significant stenosis was defined as at least 50% of one of the coronary arteries; for POAD – measuring the ankle brachial pressure index of lower extremities at the beginning of the study, with significant values below 0.90 on at least one leg; for general RF – assessment of the presence of arterial hypertension, smoking, and diabetes mellitus history; higher LDL and triglycerides; and lower HDL blood levels. Systolic and diastolic blood pressure measurements were performed at the beginning and end of the study, manually with a mercury sphyngomanometer with three consecutive readings, at least 1 week apart. The diagnosis of arterial hypertension was based on mean systolic blood pressure exceeding 140 mmHg and/or diastolic exceeding 90 mmHg. Serum lipid concentrations were measured after 12-h fasting at the beginning and end of the study. LDL was estimated using the Friedwald equation and standard assays for total cholesterol, HDL, and triglycerides. Higher values were set: LDL ≥3.5 mmol/l, triglycerides ≥1.7 mmol/l, and HDL ≤1.3 mmol/l.

## Statistical Analysis

Differences between mean values were analyzed using student t-test. Differences between degree of stenosis, plaque quality, TIA, AF, stroke, deaths, ischemic lesions on CT findings between ACS and SCS groups were established using Chi-square and Fisher test, with level of significance set at $p < 0.05$. In cases with frequency below five, we used Yates correction. Correlations between the groups of stenosis degree, plaque quality, peak systolic and end diastolic velocity, blood flow volume, and onset of neurological events and deaths frequency among the ACS and SCS group were performed using Spierman correlation (correlation coefficient = R; $p$ = level of significance), and Kruskal-Wallis one-way analysis of variance (Kruskal Wallis test value = H; $p$ = level of significance).

Stroke frequency during the follow-up was presented as stroke-free interval using Kaplan Mayer life table analysis. The diagnostic value of CDS technique was assessed in relation to results of carotid arteriography by calculations of sensitivity (Sz), specificity (Sc), and diagnostic accuracy (Ac) using the following formulas:

$$Sz = TrP/(TrP+FN) \times 100$$

$$Sc = (TrN)/(TrN+FP) \times 100$$

$$Ac = (TrP+TrN)/(ToP+ToN) \times 100$$

TrP = true positive, FP = false positive, TrN = true negative, FN = false negative, ToP = total positive (true positive + false positive), ToN = total negative (true negative + false negative).

## RESULTS

The mean age of the SCS group was significantly higher than in the ACS group (Table 1).

The analysis of the cardiovascular risk factors showed that the frequency of smoking and lower HDL level were significantly higher in the SCS vs. ACS group, while the mean values of the diastolic pressure were significantly higher in the ACS group and higher LDL level in SCS group at the beginning of the study (Table 1).

Among the risk factors, only lower HDL level showed significant association with the mix of all unstable plaque types in the ACS group (Table 2).

**TABLE 1**
**Demographics, Clinical Characteristics, and Risk Factors of ACS and SCS Groups**

| Variables [Mean ± S.D. or No. (%)] | ACS (n = 111) | SCS (n = 58) | p(χ2) |
|---|---|---|---|
| Age | 64.4 ± 9 | 67.6 ± 10 | 0.001* |
| Male | 78 (70%) | 45 (78%) | 0.310 |
| Female | 33 (30%) | 13 (22%) | 0.543 |
| Arterial hypertension | 73 (66%) | 45 (78%) | 0.112 |
| Systolic | 158.6 ± 27.7 | 165.4 ± 23.9 | 0.113* |
| Diastolic | 86.9 ± 15.5 | 80.8 ± 12.4 | 0.010* |
| Higher LDL (≥3.5 mmol/l) | 54 (49%) | 31 (53%) | 0.555 |
| LDL | 3.40 ± 0.45 | 3.61 ± 0.46 | 0.005* |
| Lower HDL (≤1.3 mmol/l) | 52 (47%) | 41 (71%) | 0.003 |
| HDL | 1.39 ± 0.31 | 1.32 ± 0.36 | 0.181* |
| Higher triglycerides (≥1.7 mmol/l) | 38 (34%) | 73 (66%) | 0.360 |
| Triglycerides | 1.74 ± 0.56 | 1.62 ± 0.59 | 0.183* |
| Diabetes mellitus | 55 (50%) | 37 (64%) | 0.077 |
| Smoking | 67 (60%) | 44 (76%) | 0.043 |
| CAD | 46 (41%) | 22 (38%) | 0.658 |
| POAD | 61(55%) | 24(41%) | 0.093 |
| ABPI | 0.84 ± 0.22 | 0.82 ± 0.24 | 0.544* |
| Renal failure | 4 (4%) | 2 (3%) | 0.935 |
| Medications after entering the study | | | |
| Acetylsalicylic acid | 101 (91%) | 54 (93%) | 0.857 |
| Clopidogrel | 34 (31%) | 15 (26%) | 0.516 |
| Statins | 108 (97%) | 57 (98%) | 0.894 |
| β-Blockers | 87 (78%) | 41 (71%) | 0.268 |
| Long-acting nitrates | 52 (47%) | 21 (36%) | 0.184 |
| ACE inhibitors | 77 (69%) | 48 (82%) | 0.059 |
| Diuretics | 28 (25%) | 23 (40%) | 0.052 |
| Calcium channel antagonists | 15 (14%) | 10 (17%) | 0.516 |
| Antidiabetics | 55(60%) | 37(64%) | 0.077 |
| CEA | 12 (11%) | 38 (66%) | 0.000 |
| All events + deaths after CEA | 0 + 1 (1%) | 8 + 0 (14%) | 0.000 |
| Total deaths | 2 (2%) | 3 (5%) | 0.453 |

* t-Test value; CAD, coronary artery disease; POAD, peripheral occlusive arterial disease; ABPI, ankle-brachial pressure index; CEA, carotid endarterectomy

CAD, POAD, and chronic renal failure frequency did not differ between the groups (Table 1).

There were no significant differences between the two groups in the aspect of number of pts taking the same medications after they entered the study. The number of carotid endarterectomy operations, and neurological events and deaths, among the patients after surgery were significantly higher in the SCS vs. ACS group (Table 1).

After 36 months, the number of asymptomatic stenoses was significantly higher in the 30–49% stenosis degree group; the same applies to symptomatic stenoses in 70–89% and over 90% stenoses groups (Table 3).

**TABLE 2**
**Association between the Risk Factors and Unstable Carotid Type Plaques**

| Risk Factors | | ACS (n = 111)* | | | | | SCS (n = 58) | | | | | | |
|---|---|---|---|---|---|---|---|---|---|---|---|---|---|
| | | ACS (n) | Mixed | *p* | Ulcer | *p* | SCS (n) | Mixed | *p* | Ulcer | *p* | IPH | *p* |
| Smoking | Yes | 67 | 14 | 0.95 | 9 | 0.22 | 44 | 21 | 0.43 | 17 | 0.23 | 4 | 0.44 |
| | No | 44 | 9 | | 2 | | 14 | 9 | | 7 | | 3 | |
| AHT | Yes | 73 | 16 | 0.66 | 7 | 0.85 | 45 | 23 | 0.86 | 16 | 0.91 | 4 | 0.86 |
| | No | 38 | 7 | | 4 | | 13 | 7 | | 8 | | 3 | |
| LDL | Yes | 54 | 14 | 0.18 | 7 | 0.46 | 31 | 15 | 0.58 | 12 | 0.88 | 3 | 0.84 |
| | No | 57 | 9 | | 4 | | 27 | 15 | | 12 | | 4 | |
| HDL | Yes | 42 | 17 | 0.0001 | 6 | 0.38 | 31 | 19 | 0.11 | 14 | 0.53 | 2 | 0.16 |
| | No | 69 | 6 | | 5 | | 27 | 11 | | 10 | | 5 | |
| DM | Yes | 55 | 12 | 0.77 | 6 | 0.72 | 37 | 16 | 0.08 | 10 | 0.38 | 5 | 0.97 |
| | No | 56 | 11 | | 5 | | 21 | 14 | | 14 | | 2 | |
| TGD | Yes | 38 | 10 | 0.60 | 4 | 0.85 | 24 | 13 | 0.75 | 10 | 0.97 | 3 | 0.74 |
| | No | 73 | 13 | | 7 | | 34 | 17 | | 14 | | 4 | |

* IPH was not taken into account because of only three cases; *p* values associated with $\chi 2$ test; AHT, arterial hypertension; DM, diabetes mellitus; TGD, triglycerides.

**TABLE 3**
**Number of Stenoses According to the Stenosis Degree after 36 Months**

| Stenosis | ACS | SCS | $\chi^2$ | *p* |
|---|---|---|---|---|
| 30–49% | 30 (27%) | 2 (3%) | 13.80 | 0.0002 |
| 50–69% | 57 (51%) | 21 (36%) | 3.52 | 0.0608 |
| 70–89% | 19 (17%) | 27 (47%) | 16.66 | 0.00001 |
| >90% | 5 (5%) | 8 (14%) | 4.63 | 0.0314 |

After 36 months, the number of new stenoses over 70% was significantly higher only in the SCS group (Table 4). After 36 months, the number of fibrous plaques was significantly higher in the ACS group; the same applies to mixed and ulcerated plaques in the SCS group (Table 5). Ulcerated plaques had a significant influence on the onset of neurological events and deaths in both groups; the same applies to mixed plaques in the ACS group (Table 6).

The number of contralateral CCA or ICA occlusions was significantly higher in the SCS group, at the beginning as well as at the end of the study (Table 7). The number of carotid stenoses with reduced carotid blood flow volume was significantly higher in the SCS group at the end of study (Table 8). Number of cases with peak systolic velocities over 130 and 210 cm/sec, as well as end diastolic velocities over 40 and 110 cm/sec, was significantly higher in the SCS than in the ACS group (Tables 9 and 10).

After 12, 24, and 36 months, the number of CVI and all neurological events was significantly higher in the SCS group, as well as number of CVI and deaths after 12 and 36 months (Table 11). During the follow-up period, the number of CVI and deaths rose significantly only in the SCS group (Table 12). Time distribution of stroke has shown a higher frequency among SCS pts (Graph 1). During the follow-up in the ACS group, the mixed plaques were significantly more frequent in pts with new neurological events, fibrous

**TABLE 4**
**Occurrence of New ≥70% Stenoses during the Follow-Up**

| Time | ACS | SCS | $\chi^2$ | *p* |
|---|---|---|---|---|
| Start | 17 (15%) | 19 (32%) | 0.78 | 0.3773 |
| 36 months | 22 (20%) | 31 (53%) | 5.06 | 0.0245 |

**TABLE 5**
**Number of Various Plaque Types at the End of Follow-Up**

| Plaque Type | ACS | SCS | $\chi^2$ | *p* |
|---|---|---|---|---|
| Calcified | 29 (26%) | 9 (16%) | 2.46 | 0.1168 |
| Fibrous | 48 (43%) | 10 (17%) | 11.43 | 0.0007 |
| Hemorrhage | 0 | 1 (2%) | 1.97 | 0.1653 |
| Mix | 30 (27%) | 38 (65%) | 23.47 | 0.00001 |
| Ulcerated | 12 (11%) | 31 (53%) | 36.59 | 0.00001 |

**TABLE 6**
**Frequency of Various Unstable Plaque Types and Their Relations to Onset of All New Neurological Events and Deaths during the Follow-Up**

| Groups | | Hemorrhage | Mixed | Ulcerated | New Events |
|---|---|---|---|---|---|
| ACS | Number | 3 | 23 | 11 | 11 |
| | H | 0.0001 | 15.87 | 16.90 | |
| | *p* | 1.0 | 0.00001 | 0.00001 | |
| SCS | Number | 7 | 30 | 24 | 23 |
| | H | 1.41 | 2.29 | 4.11 | |
| | *p* | 0.233 | 0.129 | 0.042 | |

**TABLE 7**
**Contralateral CCA or ICA Occlusion at the Beginning and End of the Study**

| Groups | Start | 36 Months |
|---|---|---|
| ACS occlusion | 2 (2%) | 3 (3%) |
| SCS occlusion | 5 (9%) | 9 (16%) |
| $\chi^2$ | 4.46 | 7.64 |
| *p* | 0.0347 | Yates 0.0057 |

**TABLE 8**
**Number of Reduced Carotid Blood Flow Cases at the Beginning and End of the Study**

| Groups | Start | 36 Months |
|---|---|---|
| ACS reduced flow | 4 (4%) | 4 (4%) |
| SCS reduced flow | 5 (9%) | 8 (14%) |
| $\chi^2$ | 1.90 | 4.55 |
| *p* | 0.167 | Yates 0.0329 |

**TABLE 9**
**Frequency of Increased Maximal Peak Systolic and End Diastolic Velocities of Examined Carotid Stenoses at the Beginning and End of the Study**

| EDV >40 cm/sec | Start | 36 Months | PSV >130 cm/sec | Start | 36 Months |
|---|---|---|---|---|---|
| ACS | 23 (21%) | 28 (25%) | ACS | 39 (35%) | 47 (42%) |
| SCS | 23 (40%) | 36 (62%) | SCS | 35 (60%) | 44 (76%) |
| $\chi^2$ | 6.89 | 21.98 | $\chi^2$ | 9.84 | 17.22 |
| *p* | 0.0087 | 0.00001 | *p* | 0.0017 | 0.0001 |

**TABLE 10**
**Frequency of Increased Maximal Peak Systolic and End Diastolic Velocities of Examined Carotid Stenoses at the Beginning and End of the Study**

| EDV >110 cm/sec | Start | 36 Months | PSV >210 cm/sec | Start | 36 Months |
|---|---|---|---|---|---|
| ACS | 4 (4%) | 9 (8%) | ACS | 11 (10%) | 12 (11%) |
| SCS | 9 (16%) | 14 (24%) | SCS | 14 (24%) | 23 (40%) |
| $\chi^2$ | 7.61 | 8.33 | $\chi^2$ | 6.12 | 19.30 |
| *p* | 0.0058 | 0.0034 | *p* | 0.0134 | 0.0001 |

were borderline significant, but calcified plaques were significantly increased in new neurological event-free pts (Table 13). At the end of the study, the total number of ischemic CT findings was significantly higher in the SCS group, and so was the number of new ischemic CT findings during the follow-up (Table 14).

Correlation of every single examined local morphologic and hemodynamic carotid plaque characteristic and ischemic CT findings on the one hand, with onset of all neurological events and deaths on the other, was significant for positive CT findings, peak systolic velocity over 210 cm/sec, peak end diastolic velocity over 110 cm/sec, stenosis ≥70%, ulcerated plaque, mixed plaque, stenosis ≥50%, and carotid blood flow reduction (Table 15).

**TABLE 11**
**Number of Neurological Events after 12, 24, and 36 Months in ACS and SCS Groups**

| Neurological Events | 12 Months | 24 Months | 36 Months |
|---|---|---|---|
| ACS TIA | 1 (1%) | 4 (4%) | 9 (8%) |
| ACS CVI | 0 | 1 (1%) | 3 (3%) |
| ACS AF | 1 (1%) | 1 (1%) | 1 (1%) |
| All events | 2 (2%) | 6 (5%) | 12 (11%) |
| SCS TIA | 5 (9%) | 10 (17%) | 20 (34%) |
| SCS CVI | 5 (9%) | 8 (14%) | 12 (21%) |
| SCS AF | 1 (1%) | 3 (5%) | 6 (11%) |
| All events | 11 (19%) | 21 (36%) | 38 (66%) |
| ACS:SCS all events | $\chi^2 = 13.26$; $p = 0.0003$ | $\chi^2 = 26.92$; $p = 0.00001$ | $\chi^2 = 52.34$; $p = 0.00001$ |
| ACS:SCS CVI | $\chi^2 = 7.23$; $p = 0.0072$* | $\chi^2 = 10.13$; $p = 0.0015$* | $\chi^2 = 13.10$; $p = 0.0003$* |

* Yates correction.

**TABLE 12**
**CVI and Death Frequency after 12 and 36 Months Follow-Up**

| Groups | 12 Months | 36 Months | $\chi 2$ | *p* |
|---|---|---|---|---|
| ACS | 2 (2%) | 5 (4%) | 0.59* | 0.4424 |
| SCS | 5 (9%) | 15 (26%) | 4.89* | 0.0270 |
| $\chi^2$ | 4.46 | 16.65* | | |
| *p* | 0.047 | 0.00001 | | |

* Yates correction; Fisher test.

**TABLE 13**
**Frequency of New Neurological Events According to ACS Plaque Types after 36 Months**

| ACS Plaque Types | Calcified | Fibrous | Hemorrhage | Mixed |
|---|---|---|---|---|
| Without symptoms | 32 | 41 | 2 | 14 |
| All neuro events | 0 | 3 | 1 | 9 |
| $\chi 2$ | 4.49 | 3.79 | 0.32 | 19.71 |
| *p* | 0.034 | 0.050 | >0.05 | 0.00001 |

**TABLE 14**
**Number of Ischemic CT Lesions in ACS and SCS at the Beginning and End of the Study**

| CT Lesions | Start | 36 Months | $\chi^2$ | *p* |
|---|---|---|---|---|
| ACS | 25 (23%) | 29 (27%) | 0.34 | 0.5315 |
| SCS | 16 (27%) | 34 (60%) | 11.39 | 0.0007 |
| $\chi^2$ | 0.53 | 17.20 | | |
| *p* | 0.4659 | 0.00001 | | |

**TABLE 15**
**Correlations of Local Risk Factors and Neurological Events and Deaths in ACS**

| ACS Risk Factors | Variance Analysis (Kruskal Wallis) | | Correlation (Spierman) | |
|---|---|---|---|---|
| | H | *p* | R | *p* |
| CT lesion | 37.55 | 0.00001 | 0.639 | 0.0000001 |
| PSV >210 cm/sec | 35.34 | 0.00001 | 0.567 | 0.0000001 |
| EDV >110 cm/sec | 25.79 | 0.00001 | 0.484 | 0.0000001 |
| Stenosis ≥70% | 19.75 | 0.00001 | 0.424 | 0.000004 |
| Plaque ulceration | 16.90 | 0.00001 | 0.392 | 0.000021 |
| Mixed plaque | 15.87 | 0.00001 | 0.380 | 0.000039 |
| Stenosis ≥50% | 11.23 | 0.0008 | 0.320 | 0.00062 |
| Reduced blood flow | 5.21 | 0.022 | 0.218 | 0.0217 |
| Contralateral occlusion | 1.19 | 0.273 | 0.104 | 0.2770 |
| Plaque hemorrhage | 0.0001 | 1.0 | / | / |

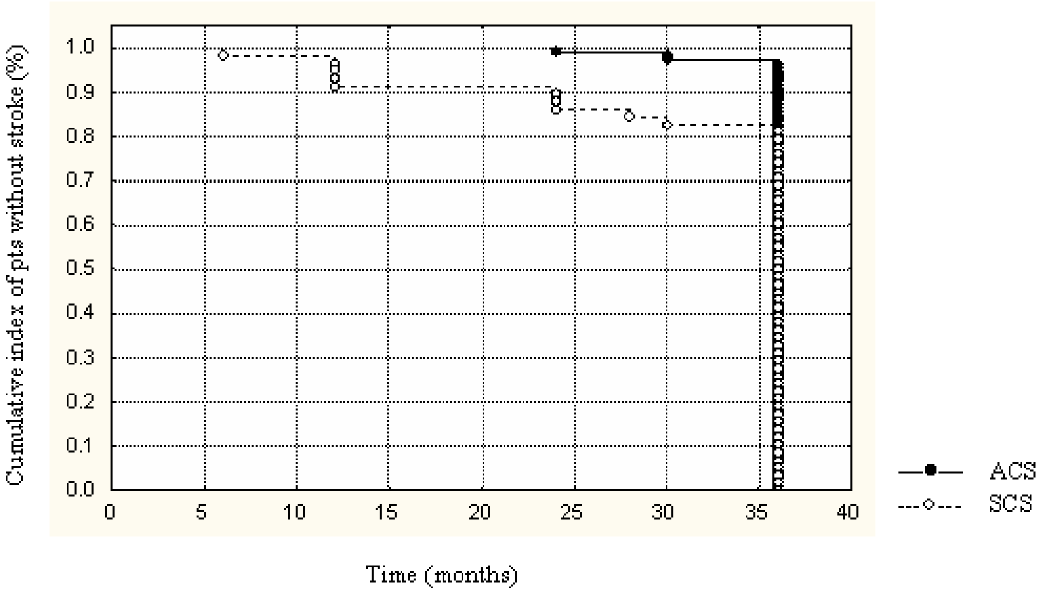

**GRAPH 1.** Stroke distribution during the follow-up period.

Referring to our carotid arteriography findings, we assessed the diagnostic value of the CDS technique in terms of carotid stenosis degree and found 101 true positive, zero true negative, one false positive, and nine false negative results. Based on these findings, we calculated that the Sz, Sc, and Ac were 92, 90, and 92%, respectively.

## DISCUSSION

The general cardiovascular risk factors are present in both carotid stenoses groups, in great extent contributing to the development of the carotid as well as coronary and peripheral artery atherosclerosis process. For most risk factors, there were no significant differences between the two groups except for the lower HDL level and smoking history frequency, as well as significantly higher LDL level in the SCS group at the beginning of the study. This could be explained by the fact that symptomatic stenoses were of high grade, with more complicated atherosclerotic plaques, which required the presence and greater influence of the main cardiovascular risk factors.

The association of all risk factors in the ACS and SCS group with plaque ulceration, mixed plaque, and intraplaque hemorrhage presence was tested respectively and the significant influence was found only for lower HDL level on mixed type plaque in the ACS group (Table 2). HDL is one of the relevant risk factors involved in the development of atherosclerosis, acting as the acceptor of cholesterol released from tissues outside the liver, transporting it back to the liver, and decreasing the risk of thrombosis. The Veterans Affairs HDL Intervention Trial provided the evidence that an increase in HDL significantly decreased the incidence of coronary heart disease–related events and stroke[27].

In the ACS group, the frequency of mild degree stenoses was significantly higher than in the SCS group, but in the SCS group, the frequency of severe stenoses and frequency of newly diagnosed stenoses ≥70% was significantly higher than in the ACS group. The increase of carotid stenosis degree is connected with atherosclerosis complications and local hemodynamic changes, which all could result in manifestation of neurological symptoms. AbuRahma et al.[8] and Norris and Zhu[9] reported that thromboembolic accidents increased with carotid stenosis degree and for stenoses ≥70%, these were 3–5% annually, but for stenoses below 50%, it is minimal[8,9]. Among the 75–90% stenoses, unstable plaque was more frequent and associated with a higher risk of thromboembolic accidents[9]. Takaya et al. found that pts who initially had an asymptomatic 50–79% carotid stenosis were frequently associated with occurrence of subsequent cerebrovascular events[10].

In the SCS group, the mixed and ulcerated type plaques were significantly more frequent than in the ACS group, but the fibrous type plaque was significantly more frequent in the ACS group. Plaque ulceration is a source of microthrombosis and subsequent embolization resulting in symptom manifestation. Hipoechoic plaque often presents an intraplaque hemorrhage, which can result in fibrous cap rupture and plaque ulceration. Fisher et al.[11] reported 34% cases with ulcerated plaques in pts with neurological symptoms, in comparison to 14% in asymptomatic pts; Mathiesen et al.[12] found that echo-rich plaques carry a lower risk of neurological symptoms. Thromboembolic events are significantly more frequent among the intraplaque hemorrhage, mixed, and ulcerated type of plaques than among fibrous and calcified plaques[13,14,15]. Steinke et al. studied a group of the severe stenoses (>80%), and found significantly more ulcerated plaques in the symptomatic (43%) than in asymptomatic stenoses (23%), but fibrous plaques were significantly more common in asymptomatic (46%) than in symptomatic stenoses (29%)[16]. Mayor et al. studied moderate and high-grade isoechoic and hyperechoic stenoses, and reported a significantly lower frequency of microembolic signals than in anechogenic and hipoechogenic lesions[17].

The number of CCA or ICA contralateral occlusions was significantly higher in the SCS group and showed that this risk factor importantly contributes to the onset of neurological symptoms. Some authors reported that the prevalence of contralateral CCA or ICA occlusion is about 5–8%, and is associated with triple accumulated relative risk for CVI[18].

Hemodynamically reduced blood flow on the affected side was more frequent in the SCS group due to the presence of more severe-degree stenoses in this group. Fowl et al. showed that the prevalence of hemodynamically significant carotid stenoses in ACS pts was below 6%[19]. Powers reported that poor collateral circulation contributes to focal cerebral symptoms[20]. High flow velocity at the site of stenosis was more frequent in symptomatic than in asymptomatic cases. The significant rise of peak flow velocity is a consequence of high-degree carotid stenosis. The jet of flow at the site of maximal stenosis can produce intimal damage and formation of microthrombi, influencing the new symptom onset[10].

Ulcerated plaques in both ACS and SCS groups significantly correlate ($p < 0.0001$ and $p < 0.05$, respectively) with the onset of all neurological events. New neurological events were more frequent in the SCS than in the ACS group during the follow-up. Ballota et al. found a statistically higher incidence of ulcerated plaques among the symptomatic ($p < 0.01$) than asymptomatic stenoses[21]. Wilterdink and Easton studied carotid stenoses over 50% and reported 1.3% CVI incidence for asymptomatic pts and 2.2–9% for the symptomatic pts[22].

Various plaque types have a different influence on the onset of new neurological events. In the ACS group, calcified and fibrous plaques ($p < 0.05$ and $p = 0.05$, respectively) are among plaques that remained asymptomatic. The most frequent new symptoms were reported in the mixed plaque group ($p < 0.0001$). This complies with results of Nandalur et al., who found that calcified plaques were 21 times less likely to be symptomatic than the noncalcified plaques ($p < 0.05$)[23]. Tegos et al. found that asymptomatic stenoses have been predominantly fibrous, while in symptomatic cases, mixed and ulcerated plaques prevailed, the latter often being involved in endoluminal thrombi growth and thus a source of embolization[24,25].

In both ACS and SCS groups, the number of CVI and deaths increased after 12 and 36 months, but significantly more in the SCS than ACS group ($p < 0.05$ and $p < 0.0001$, respectively). Tegos et al. and Kalogeropoulos et al. found that symptomatic stenoses were more often of high degree and unstable type, and thus associated with more frequent onset of new neurological events[24,26].

## CONCLUSION

This study suggests that among the local morphologic and hemodynamic characteristics of the carotid plaque, significant correlation in predicting the neurological events onset and deaths was shown for stenosis degree over 70%, ulcerated type plaque, mixed type plaque, increased peak systolic velocity over 210 cm/sec, increased end diastolic flow velocity over 110 cm/sec, stenosis degree over 50%, and reduced blood flow volume at the stenosis site. Among the general cardiovascular risk factors, only lower HDL level significantly correlated with presence of the carotid mixed type plaque in the ACS group.

## References

1. National Heart, Lung and Blood Institute (2006) Incidence and Prevalence: 2006 Chart Book on Cardiovascular and Lung Diseases. Bethesda, MD.
2. Venkatachalm, L., Bobinac Georgievski, A., Al Yazeedi, W., Singh, R., and Uribazo Garrido, H. (2008) Length of stay in in-patient rehabilitation after stroke in Quatar. *TheScientificWorldJOURNAL: TSW Child Health & Human Development* **8**, 547–555.
3. Bicigaluppi, M. and Hermann, D.M. (2008) New targets of neuroprotection in ischemic stroke. *TheScientificWorldJOURNAL* **8**, 698–712.
4. American Heart Association (2008) Heart Disease and Stroke Statistics-2008 Update Dallas, Texas. Chap. 4, p. 15.
5. Naderishvili, Z.G., Rothwell, P.M., Beletsky, V., Pagniello, A., and Norris, J.W. (2002) Long term risk of stroke and other vascular events in patients with asymptomatic carotid artery stenosis. *Arch. Neurol.* **59**, 1162–1166.
6. Thomson, J.E. (1991) Carotid endarterectomy for asymptomatic carotid stenosis: an update. *J. Vasc. Surg.* **13**, 669–676.
7. Flanigan, P.D. (1989) Carotid endarterectomy without arteriography: can duplex alone suffice? *Semin. Vasc. Surg.* **2**, 18–23.

8. AbuRahma, A.F., Wulu, J.T., and Crofty, B. (2002) Carotid plaque ultrasonic heterogenity and severity of stenosis. *Stroke* **33**, 1772–1775.
9. Norris, J.W. and Zhu, C.Z. (1992) Silent stroke and carotid stenosis. *Stroke* **23**, 483–485.
10. Takaya, N., Yuan, C., Chu, B., Saam, T., Underhill, H., Cai, J., Tran, N.L., Polisar, N.L., Isaac, C., Ferguson, M.S., Garden, G.A., Cramer, S.C., Maravilla, K.R., Hashimoto, B.H., and Atsukami, T.S. (2006) Association between carotid plaque characteristics and subsequent ischemic cerebrovascular events: a prospective assessment with MRI-initial results. *Stroke* **37,** 818–823.
11. Fisher, M., Paganini-Hill, A., Martin, A., Cosgrove, M., Toole, J.F., Barnett, H.J., and Norris, J. (2005) Carotid plaque pathology: thrombosis, ulceration, and stroke pathogenesis.. *Stroke* **36**, 253–257.
12. Mathiesen, E.B., Bonaa, K.H., and Joakimsen, O. (2001) Echolucent plaques are associated with high risk of ischemic cerebrovascular events in carotid stenosis: the Tromso study. *Circulation* **103**, 2171–2175.
13. Sabetai, M.M., Tagos, T.J., Nicolaides, A.N., El-Atrazy, T.S., Dhanjil, S., Griffin, M., Belcaro, G., and Geroulacos, G. (2000) Hemispheric symptoms and carotid plaque echo morphology. *J. Vasc. Surg.* **31(1)**, 39–49.
14. Tegos, T.J., Kalomiris, K.J., Sabetai, M.M., Kalodiki, E., and Nicolaides, A.N. (2001) Significance of sonographic tissue and surface characteristics of carotid plaques. *AJNR Am. J. Neuroradiol.* **22**, 1605–1612.
15. Gao, P., Chen, Z.-Q., Bao, Y.-H., Jiao, L.-Q., and Ling, F. (2007) Correlation between carotid intraplaque hemorrhage and clinical symptoms. Systematic review of observational studies. *Stroke* **38**, 2382–2390.
16. Steinke, W., Hennerici, M., Rautenburg, W., and Mohl, J.P. (1992) Symptomatic and asymptomatic high grade stenoses in doppler color flow imaging. *Neurology* **42**, 131–138.
17. Mayor, I., Comelli, M., Vassileva, E., Burkhard, P., and Sztajel, R. (2003) Miocroembolic signals and carotid plaque morphology: a study of 71 patients with moderate or high grade carotid stenosis. *Acta Neurol. Scand.* **108**, 114–117.
18. Barnett, H. (1993) Lessons from symptomatic carotid stenosis of interest in considering asymptomatic disease. In *Cerebral Revascularisation*. Bernstein, E., Callow, A., Nikolaides, A., and Shiffrin, E., Eds. Med-Orion Publishing, London. pp. 483–487.
19. Fowl, R.J., Marsch, J.G., Love, M., Patterson, R.B., Shukla, R., and Kempczinski, R.F. (1991) Prevalence of hemodynamically significant stenosis of the carotid artery in an asymptomatic veteran population. *Surg. Gynecol. Obstet.* **172(1)**, 13–26.
20. Powers, W.J. (1991) Cerebral hemodynamics in ischemic cerebrovascular disease. *Ann. Neurol.* **29**, 231–240.
21. Ballota, E., Da Gian, G., and Renon, L. (2000) Carotid plaque gross morphology and clinical presentation: a prospective study of 457 carotid artery specimens. *J. Surg. Res.* **89,** 78–84.
22. Wilterdink, J.L. and Easton, J.D. (1992) Vascular event rates in patients with atherosclerotic cerebrovascular disease. *Arch. Neurol.* **49**, 857–863.
23. Nandalur, K., Baskurt, E., Hagspiel, K.D., Phillips, C.D., and Cramer, C.M. (2005) Calcified carotid atherosclerotic plaque is associated less with ischemic symptoms than is noncalcified plaque on MDCT. *AJR Am. J. Roentgenol.* **184,** 295–298
24. Tegos, T.J., Sabetai, M.M., Nicolaides, A.N., Pare, G., Elatrozy, T.S., Dhanjil. S., and Griffin, M. (2000) Comparability of the ultrasonic tissue characteristics of carotid plaques. *J. Ultrasound Med.* **19**, 399–407.
25. Tegos, T.J., Sohail, M., Sabetai, M.M., Robless, P., Akbar, N., Pare, G., Stensby, G., and Nicolaides, A.N. (2000) Echomorphologic and histopathologic characteristics of unstable carotid plaques. *AJNR Am. J. Neuroradiol* . **21**, 1937–1944.
26. Kalogeropoulos, A., Terzis, G., Chrysanthopoulou, A., Hahalis, G., Siabis, D., and Alexopoulos, D. (2006) Risk for TIA is mainly determined by intima-media thickness and carotid plaque echogenicity. *Atherosclerosis* **12**, 190–196.
27. Rubins, H.B., Robins, S.J., Collins, D., Andersen, J.W., Elam, M.B., Fass, F.H., Linares, E., Schaefer, E.J., Schectman, G., Wilt, T.J., and Wittes, J.T. (1999) Gemfibrozil for the secondary prevention of coronary heart disease in men with low levels of high-density lipoprotein cholesterol. *N. Engl. J. Med.* **341,** 410–418.
